# Response to dexamethasone is glucose-sensitive in multiple myeloma cell lines

**DOI:** 10.1186/1756-9966-30-81

**Published:** 2011-09-13

**Authors:** Ellen Friday, Johnathan Ledet, Francesco Turturro

**Affiliations:** 1 Feist-Weiller Cancer Center, Louisiana State University Health Sciences Center, Shreveport, Louisiana, USA; 2 Department of Lymphoma/Myeloma, Unit 429, MD Anderson Cancer Center, 1515 Holcombe Blvd, Houston, Texas 77030 USA

## Abstract

**Background:**

Hyperglycemia is among the major side effects of dexamethasone (DEX). Glucose or glucocorticoid (GC) regulates the expression of thioredoxin-interacting protein (TXNIP) that controls the production of reactive oxygen species (ROS) through the modulation of thioredoxin (TRX) activity.

**Methods:**

Multiple myeloma (MM) cells were grown in 5 or 20 mM/L glucose with or without 25 μM DEX. Semiquantitative reverse transcription-PCR (RT-PCR) was used to assess TXNIP RNA expression in response to glucose and DEX. ROS were detected by 5-6-chloromethyl-2',7'-dichlorodihydrofluorescein diacetate (CM-H2DCFDA). TRX activity was assayed by the insulin disulfide-reducing assay. Proliferation was evaluated using CellTiter96 reagent with 490-nm absorbtion and used to calculate the DEX IC_50 _in 20 mM/L glucose using the Chou's dose effect equation.

**Results:**

TXNIP RNA level responded to glucose or DEX with the same order of magnitude ARH77 > NCIH929 > U266B1 in these cells. MC/CAR cells were resistant to the regulation. ROS level increased concurrently with reduced TRX activity. Surprisingly glucose increased TRX activity in MC/CAR cells keeping ROS level low. DEX and glucose were lacking the expected additive effect on TXNIP RNA regulation when used concurrently in sensitive cells. ROS level was significantly lower when DEX was used in conditions of hyperglycemia in ARH77/NCIH9292 cells but not in U266B1 cells. Dex-IC_50 _increased 10-fold when the dose response effect of DEX was evaluated with glucose in ARH && and MC/Car cells

**Conclusions:**

Our study shows for the first time that glucose or DEX regulates important components of ROS production through TXNIP modulation or direct interference with TRX activity in MM cells. We show that glucose modulates the activity of DEX through ROS regualtion in MM cells. A better understanding of these pathways may help in improving the efficacy and reducing the toxicity of DEX, a drug still highly used in the treatment of MM. Our study also set the ground to study the relevance of the metabolic milieu in affecting drug response and toxicity in diabetic versus non-diabetic patients with MM.

## Background

Despite the booming of novel agents for the treatment of multiple myeloma (MM) such as proteasome inhibitor bortezomib, and immuno-modulator agents thalidomide or lenalidomide, dexamethsone (DEX) remains one of the most active agents in the treatment of this disease [1]. In fact, most of the combinations with the novel agents still include DEX as a backbone [1]. Furthermore, single agent DEX has represented the control arm in the studies that have assessed efficacy and safety of the novel agent combinations [2,3]. Although the efficacy of DEX-based combinations has been widely proven, DEX is associated with notable toxicity either as single agent or in combination with novel agents. A recent study has shown similar efficacy but with less toxicity by reducing the dose of DEX in combination with the novel agent lenalidomide [4]. Hyperglycemia is among the major side effects of DEX and none of the studies has addressed the question whether the action of DEX is different in condition of hyperglycemia versus normoglycemia in treated MM patients. We have previously shown that hyperglycemia regulates thioredoxin (TRX) activity-reactive oxygen species (ROS) through induction of thioredoxin-interacting protein (TXNIP) in metastatic breast cancer-derived cells MDA-MB-231 [5]. We also showed that hyperglycemia-regulated TXNIP-ROS-TRX axis was relevant for the response of MDA-MB-231 cells to paclitaxel cytotoxicity [6]. Based on both observations that DEX induces hyperglycemia and that hyperglycemia may interfere with the cell response to drugs, we investigated the axis TXNIP-ROS-TRX in conditions of increased level of glucose (e.g., mimicking *in *vivo conditions of hyperglycemia) and in response to DEX in a pool of cells derived from multiple myeloma. Our results set the track for further investigating the relevance of metabolic conditions of the patients with multiple myeloma and response to therapy.

## Materials and methods

### Cell lines and tissue culture

Multiple myeloma-derived cell lines NCIH929, ARH77, U266B1 and MC/CAR were purchased from American Type Culture Collection (Manassas, VA). Dexamethasone and phloretin were purchased from Sigma-Aldrich (St. Louis, MO) Cells were routinely cultured in RPMI1640/10%FBS/5 mM glucose. For chronic hyperglycemia conditions, cells were chronically grown in RPMI 1640/10% FBS containing 20 mM glucose. For dexamethasone response cells were cultured in either 5 or 20 m chronically and dexamethasone (25 uM) added to media for 24 hours prior to harvest. Glucose uptake inhibition studies were accomplished by adding phloretin (200 uM) to media and cells harvested after 24 hours.

### TXNIP RT-PCR, ROS assay and TRX activity

All experiments were run in triplicate for analysis. Cells were harvested and each sample split into three aliquots for RNA isolation, ROS and TRX activity analysis. Total RNA was isolated using Aquapure RNA isolation kit (Bio-Rad, Hercules, CA) and first strand c-DNA synthesis by iScript c-DNA amplification kit (Bio-Rad) according to manufacture's protocol. Primers and PCR conditions were as previously described [5]. We have previously shown that increased RNA correlates with level of TXNIP protein [5]. ROS were detected by 5-6-chloromethyl-2', 7'-dichlorodihydrofluorescein diacetate (CM-H2DCFDA) and measured for mean fluorescence intensity by flow cytometry as previously described [5]. TRX-activity was assessed by the insulin disulfide assay as previously described [5]. Fold-change (> 1 versus < 1 fold increase/decrease, 1 = no change) was obtained for each cell line. Cell lines which showed response (NCIH929, ARH77, U266B1) were further grouped and compared to non-responsive MC/CAR cell line.

#### Dexamethasone IC_50 _calculation

IC 50 were calculated by the method of Chou and Talalay using Calcusyn software (Biosoft, Cambrigdge UK)

### Statistical analysis

Differences between treatments were evaluated by ANOVA or student's t-test and accepting as significant differences if p < 0.05.

## Results

### Differences in TXNIP-ROS-TRX axis-response to hyperglycemia in MM cells

**Figure 1:**
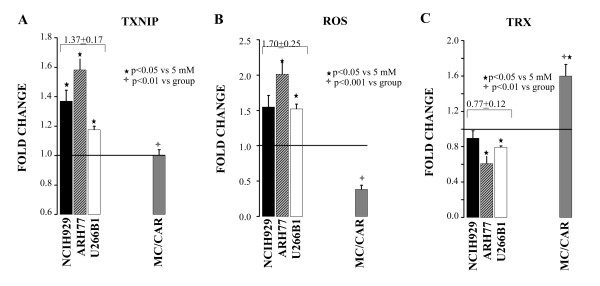
**Txnip -ROS- TRX axis regulation by hyperglycemia varies among cell lines**. Cells were grown chronically in RPMI 5 or 20 mM glucose (GLC). Data is represented as fold change over 5 mM baseline, with > 1 fold change indicating an increase over baseline and < 1 a decrease over baseline levels. Multiple myeloma-derived ARH77, NCIH929 and U266B1, which showed glucose response, were grouped and the mean value ± SD for the group presented above.. A. Thioredoxin-interacting protein (TXNIP) RNA levels. B. Reactive l oxygen species (ROS)-levels. C.Thioredoxin (TRX) activity. Black star represents p-value compared to 5 mM, cross indicates p- value of MC/CAR compared to grouped value.

We assessed the TXNIP RNA level, ROS production and TRX activity in response to isolated hyperglycemia. The function of TXNIP as a modulator of the redox system through the binding of the TRX active cysteine residues has been elucidated [7,8]. Furthermore, the promoter region of the TXNIP gene contains carbohydrate responsive elements (ChoRE) conferring the responsiveness of the gene directly to glucose [9,10]. We have also recently shown that there is strong correlation between TXNIP RNA and TXNIP protein level to justify our decision to assess only RNA levels in the cells [5]. Hyperglycemia [20 mM versus 5 mM glucose] significantly affected the fold-change of increased levels of TXNIP RNA level (mean 1.37 ± 0.17) and ROS level (mean 1.70 ± 0.25) in NCIH9292, ARH77 and U266B1 cells (Figure 1A). As expected TRX activity concurrently declined an average of 0.77 ± 0.12 in the same cell lines (Figure 1C). Unexpectedly, glucose induced an increase in TRX activity (1.6 ± 0.13 fold) associated with decreased ROS activity (0.38 ± 0.06 fold), and unchanged TXNIP RNA level in MC/CAR cells (Figure 1A-C). These results clearly show that TXNIP RNA regulation by hyperglycemia varies among multiple myeloma cell lines with a grading in response ARH77 > NCIH929 > U266B1 as compared to non-responder MC/CAR cells (Figure 1A-C). This effect translates in a consequent grading of reduced TRX activity and increased ROS level by the same order in these cell lines. On the other hand, hyperglycemia seems to have a protective effect by increasing TRX activity and reducing ROS level in MC/CAR cells, the ones not responding to glucose-TXNIP regulation. This effect hampers ROS production in the same cell line.

### Response of the TXNIP-ROS-TRX axis to DEX in conditions of hyperglycemia

**Figure 2:**
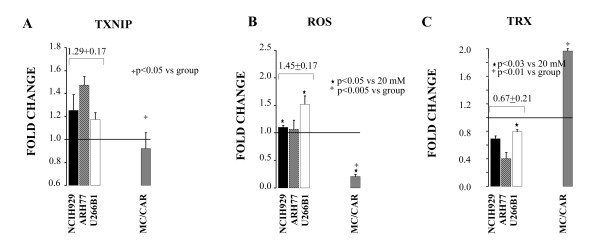
**Hyperglycemia and dexamethasone (DEX) do not have an additive effect on TXNIP-ROS-TRX**. Cells were grown in 20 mM glucose (GLC) ± dexamethasone (25 μM) (DEX) for 24 h. Data is represented as fold change over 20 mM baseline, with > 1 fold change indicating an increase over baseline and < 1 a decrease over baseline levels. Multiple myeloma-derived ARH77, NCIH929 and U266B1, which showed dex response, were grouped and the mean value ± SD for the group presented above. A. Thioredoxin-interacting protein (TXNIP) RNA levels. B. Reactive oxygen species (ROS)-levels. C.Thioredoxin (TRX) activity. Black star represents p-value compared to 20 mM GLC alone, cross indicates p- value of MC/CAR compared to grouped value.

DEX induces hyperglycemia by itself as adverse event in some patients. Furthermore, recent studies have demonstrated that TXNIP gene contains glucocorticoid-responsive elements (GC-RE) and it has been described as prednisolone-responsive gene in acute lymphoblastic leukemia cells [11,12]. We decided to study the response of TXNIP-ROS-TRX axis *in vitro *as a mimicker of the *in vivo *situation involving a patient who either experiences GC-induced hyperglycemia or uses DEX in a condition of existing frank diabetes. Our expectations were that DEX would have had an additive effect on the axis amplifying the ROS production and the oxidative stress. When DEX was added to cells grown in condition of hyperglycemia, no additive effect was seen in NCIH929, ARH77 and U266B1 cell lines. The mean TXNIP response was similar with DEX (mean 1.29 ± 0.17) or without it (mean 1.37 ± 0.19) in the same three cell lines (e.g., compare Figure 1A and 2A). ROS levels were significantly lower as compared to isolated hyperglycemia in NCIH929 and ARH77 cells but unchanged in U266B1 (Figure 1B and 2B). TRX activity was not different compared to isolated hyperglycemia in all three-cell lines (Figure 1C and 2C). Paradoxically, the data suggested that DEX was hampering the effect of TXNIP on ROS level in NCIH929 and ARH77 cells, but not in U266B1 cells that were less sensitive to TXNIP-ROS-TRX axis regulation in the first place. More interestingly DEX significantly decreased ROS level (0.38 ± 06 vs 0.21 ± 0.04, p < 0.05) in MC/CAR cells (Figure 1B and 2B). This event was associated with an increase, though not significantly different, of TRX activity (1.97 ± 0.12 vs 1.60 ± 0.13, p = 0.07) in the DEX-treated MC/CAR cells (Figure 1C and 2C). These findings suggested that DEX was also playing a protective effect from ROS production in hyperglycemia TXNIP-TRX insensitive MC/CAR cells implying the involvement of a different biochemical milieu in these cells.

### TXNIP is DEX responsive gene in some MM cells but not in others

**Figure 3:**
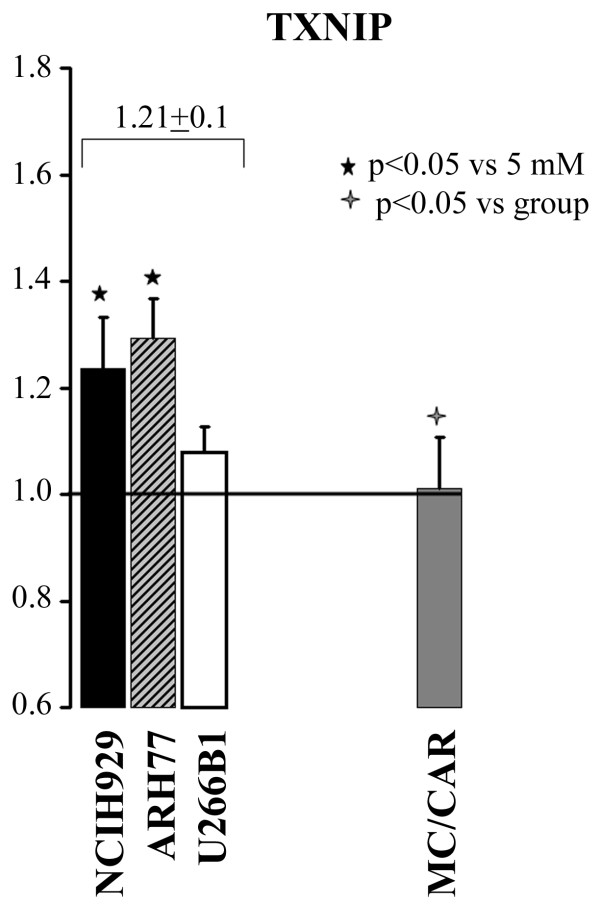
**TXNIP is DEX responsive in some MM cell lines but not others**. Cells were grown in 5 mM glucose (GLC) ± dexamethasone (25 μM) (DEX) for 24 h. Data is represented as fold change over 5 mM baseline, with > 1 fold change indicating an increase over baseline and < 1 a decrease over baseline levels. Multiple myeloma-derived ARH77, NCIH929 and U266B1, which showed dex response, were grouped and the mean value ± SD for the group presented above. Black star represents p-value compared to 5 mM GLC alone, cross indicates p- value of MC/CAR compared to grouped value.

Based on the literature saying that TXNIP gene is responsive to GC we expected an additive effect of DEX and glucose on its expression [11,12]. Surprisingly, our data were opposing this expectation making us wondering whether TXNIP gene would have responded to DEX in MM cells in the first place. For this purpose, we treated cells with DEX in conditions of normoglycemia (5 mM). TXNIP RNA significantly increased in NCIH929 and ARH77 cells, less in U266B1 cells and definitively remained unchanged in MC/CAR (Figure 3). DEX-mediated TXNIP RNA level overlapped the same pattern seen with glucose response in the same cell lines: ARH77 > NCIH929 > U266B1. These data suggest that glucose and DEX-mediated TXNIP regulation may share the same regulatory mechanism that varies in MM cells to the point of absolute unresponsiveness as observed in MC/MCAR cells. Furthermore, DEX directly increased TRX actitvity and ROS level in MC/CAR cells grown in 5 mM glucose (data not shown).

### Cellular level of glucose regulates TXNIP RNA levels and ROS in ARH77 cells

**Figure 4:**
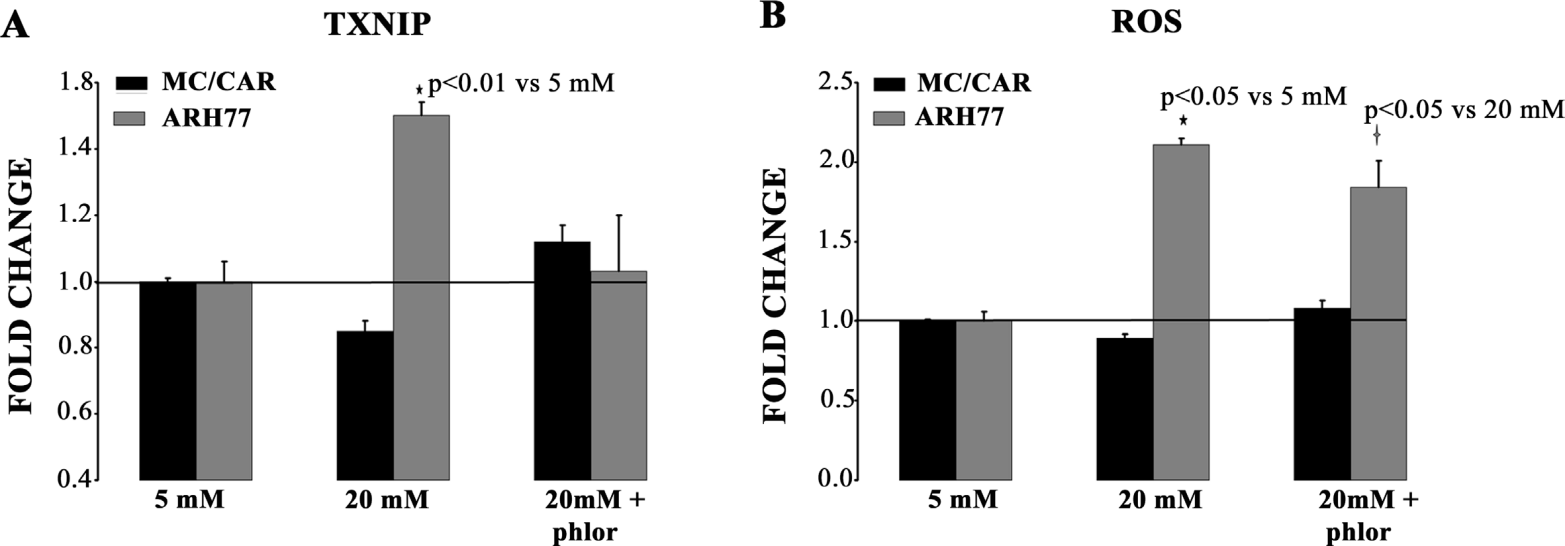
**A. Blocking glucose transport blocks the hyperglycemia effect oon thioredoxin-interacting protein (TXNIP) RNA levels**. Cells were grown in 5 mM glucose or 20 mM chronically.. For glucose uptake inhibition, phlor (200 μM) was added to 20 mM media and cells harvested after 24 hours. Fold change is based on comparison to 5 mM glucose. B. Reactive oxygen species (ROS)-levels in response to phlor pre-treatment. Cells were treated as in A. ROS levels were measured as mean fluorescence of 50,000 cells and compared to 5 mM as baseline.

To assess whether the glucose-induced increase of TXNIP RNA and ROS level were regulated by the intracellular level of glucose, we inhibited the transport of the glucose with phloretin which is an effective though not specific inhibitor of GLUT1 transporter as previously shown [5]. For this purpose, we investigated ARH77 cells that had shown the highest TXNIP RNA level response compared to the unresponsive MC/CAR cells (Figure 1A). As expected, phloretin blocked the hyperglycemia effect on TXNIP RNA level (1.5 ± 0.05 vs. 1.03 ± 0.03, p < 0.01) (Figure 4A) and significantly reduced ROS (2.1 ± 0.08 vs 1.84 ± 0.14, p < 0.05) in ARH77 cells (Figure 4B). The addition of phloretin had no effect on either TXNIP or ROS levels in the MC/CAR cells (Figure 4A, B). This confirmed that glucose played a major role in the TXNIP RNA regulation in responsive cells ARH77.

### Hyperglycemia increases the DEX-IC_50 _in MM cells

**Figure 5:**
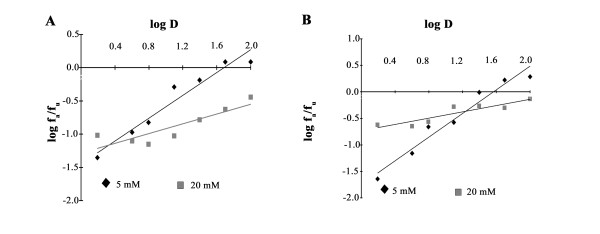
**Hyperglycemia increase the DEX-IC_50 _in MM cells **. Cells were grown in 5 or 20 mM glucose chronically. Dexamethasone, in varying concentrations, was added for 24 hour after which cells were harvested. IC50 was calculated using Calcusyn software and represented as median dose response. A. ARH77 response B. MC/CAR response.

At this point our data were suggesting that DEX and glucose together reduced ROS production in ARH77, NCIH929 and MC/CAR cells independently from the TXNIP-TRX regulation. Paradoxically, DEX + glucose further decreased ROS level by increasing TRX activity in MC/CAR cells. It seemed that DEX was mitigating the oxidative stress and ROS production induced by glucose in those cells independently from TXNIP expression. We then decided to test the hypothesis of TXNIP-independent effect by assessing the cytotoxicity of DEX in TXNIP-glucose/DEX responsive cells ARH77 and TXNIP-glucose/DEX unresponsive cells MC/CAR. When the dose response effect to DEX was evaluated in ARH77 and MC/CAR cells in 20 mM glucose, we found that hyperglycemia increased the IC_50 _for both cell lines by a factor of 10 (ARH77: 48 μM to 510 μM; MC/CAR 36 μM to 303 μM) (Figure 5). These data suggest that MM cells were more resistant to DEX in conditions of hyperglycemia, probably because of the hampering effect of DEX on ROS production as shown in Figure 2.

## Discussion

Our study addresses the response of cancerous cells in conditions of hyperglycemia either related to drug induction or underlining diabetes. More specifically, the study addresses the question on how cancerous cells handle the excess of glucose that a drug as part of the treatment or the deranged metabolism of the host may cause. We used a cell model derived from MM because this disease affects middle aged or older patients who present a higher incidence of diabetes and are treated with combinations of drugs that include a GC [1]. DEX as an example of GC induces hyperglycemia either in situations of normal glycemia or even in case of diabetes under insulin therapy or oral antidiabetic drugs. Therefore, the use of the drug may pose cancerous cells in metabolic situations the consequences of which onto the response to the treatment with it are unknown. We have recently shown that glucose regulates ROS production through TXNIP regulation and TRX activity in breast cancer derived cells [5,6]. TXNIP is also regulated by GC and is one of the genes that predicts apoptotic sensitivity to GC as recently shown in the gene expression profiling of leukemic cells and primary thymocytes [13]. We show that TXNIP-ROS-TRX axis is functional in response to glucose in 3 out of 4 MM cell lines tested and TXNIP RNA level is responsive to DEX in the same 3 cell lines. Although the metabolic axis responds to glucose or DEX with a various magnitude, this is completely unresponsive in U266B1 cell line. Our data suggest that TRX activity might be directly regulated by glucose or DEX in these cells that have unchanged levels of TXNIP RNA, a major endogenous inhibitor of TRX activity [14]. The direct regulation of TRX activity by glucose has been described in diabetic rat heart but never in cancerous cells [15]. Thioredoxin reductase 1, a major regulator of TRX oxidation, is GC-sensitive as shown in epithelial cells [16]. Although we have not investigated the mechanism in MM cells U266B1, we speculate that the metabolic conditions triggered by an excess of glucose or directly by DEX activates the TRX system to scavenger the excess of ROS that would have otherwise occurred, particularly when TXNIP is downregulated. Obviously, this point needs to be proven in future studies.

Gatenby and Gilles have recently described the dependence of highly proliferative cancerous cells upon aerobic glycolysis [17]. This acquired phenotype highly depends on persistent glucose metabolism to lactate in conditions of hypoxia [17]. We have shown that the shift to lactate metabolism in excess of glucose is associated with increased levels of TXNIP protein that increases ROS levels through inhibition of TRX activity in breast cancer derived cells MDA-MB-231 [5,6]. We show for the first time that a similar mechanism operates in some MM cell lines at various degree of efficiency. We also show for the first time that the same MM cells respond to DEX-mediated TXNIP regulation. Surprisingly, we also observe a glucose-sensitive response of MM cells to DEX that makes the cells less susceptible to the cytotoxic effects of the drug. This observation was unexpected because we anticipated that TXNIP regulation would have been enforced by the combination of glucose and DEX both containing responsive elements in the regulatory part of TXNIP gene. In fact, glucose or DEX was individually able to exert TXNIP regulation at various degrees in responsive cells. Their effect was though not augmented by the combined exposure of the cells as expected. One possible explanation might be that ChoRE and GC-RE are competing with each other or that the action of DEX prevails on the glucose by mechanism directly interfering with ROS production outside the nucleus in those MM cells, ARH77 and MC/CAR. Obviously, the speculation portends further work in support of the hypothesis. Furthermore, DEX and glucose may exert their effects outside the nucleus at the level of mitochondria where ROS are mainly produced. In fact, evidence suggests that TXNIP triggers activation of nuclear transcription regulation by MondoA at the mitochondrial level, which favors cross talk between mitochondria and nucleus [18,19]. Emerging pathways of non-genomic GC signaling involving direct action of GC on the mitochondria have been recently described in T cells and neurons [20,21]. Although a recent study has shown that DEX-induced oxidative stress enhances radio-sensitization of MM cells, this effect was not studied in conditions of hyperglycemia [22].

## Conclusions

In conclusion, although our study elucidates never-described before regulation of glucose and DEX of important components of ROS regulation through TXNIP modulation or direct interference with TRX activity, we are well aware of the limitations of the study itself. First our study is a very preliminary study that originates hypothesis and consider the relevance of the metabolic conditions of the host (diabetes, hyperglycemia, etc) rather than the relevance of diabetes as a cause of malignance. Whether this has consequences on the response to therapy or not needs to be assessed. Second, our study lacks both the elucidation of the mechanisms underlying our observation and the validation of the observation itself in cells directly and freshly isolated from patients. The easy way to validate the concept will be to analyze survival and disease free survival/end points retrospectively in patients with multiple myeloma treated with DEX in conditions of hyperglycemia versus normal glycemia. Despite the limitation that EBV-infected cell lines (ARH-77 and MC/CAR) may pose as results and the fact that normal control cell counterparts are lacking in our study, we still believe that we represent a grading of response in the four cell lines tested that reflect the heterogeneity of cells undergone malignant transformation. For the first time, we show that glucose modulates the activity of DEX and this action seems mainly involving pathways regulating ROS in MM cells. Whether this finding will help in reducing DEX toxicity or improving its efficacy particularly in combination with other agents remains unclear. A better understanding of these pathways may help in improving the efficacy and reducing the toxicity of DEX, a drug still highly used in the treatment of MM. Our study also set the ground to study the relevance of the metabolic milieu in affecting drug response and toxicity in diabetic versus non-diabetic patients with MM

## Abbreviations

DEX: dexamethasone; GC: glucocorticoid; TXNIP: thioredoxin interacting protein; TRX: thioredoxin; MM: multiple myeloma; IMDs: immune modulator drugs; RT-PCR: reverse transcriptase polymerase chain reaction; CM-H2DCFDA: 5-6 chloromehtyk-2-7-dichloridihydrofluorescien diacetate; ROS: reactive oxygen species; ChoRE: carbohydrate responsive elements; GC-RE: glucocorticoid responsive element; IC_50_: inhibitory concentration 50%.

## Competing interests

FT has served as Advisory Board member for Celgene, Millennium Pharmaceuticals and received research funding from Merck Oncology. EF and JL report no competing interests.

## Authors' contributions

FT originated the idea of the project. EF defined the experimental plan and executed with JL's help. FT and EF drafted the manuscript and finalized it. All authors read and approved the final manuscript
